# A Mouse Model for Juvenile, Lateral Fluid Percussion Brain Injury Reveals Sex-Dependent Differences in Neuroinflammation and Functional Recovery

**DOI:** 10.1089/neu.2019.6675

**Published:** 2020-02-04

**Authors:** Elizabeth A. Newell, Brittany P. Todd, Zili Luo, Lucy P. Evans, Polly J. Ferguson, Alexander G. Bassuk

**Affiliations:** ^1^ Department of Pediatrics, University of Iowa, Iowa City, Iowa.; ^2^ Medical Scientist Training Program, University of Iowa, Iowa City, Iowa.

**Keywords:** age-dependent, FPI, function, neuroinflammation, sex

## Abstract

Traumatic brain injury (TBI) is a leading cause of death and disability that lacks targeted therapies. Successful translation of promising neuroprotective therapies will likely require more precise identification of target populations through greater study of crucial biological factors like age and sex. A growing body of work supports the impact of these factors on response to and recovery from TBI. However, age and sex are understudied in TBI animal models. The first aim of this study was to demonstrate the feasibility of lateral fluid percussion injury (FPI) in juvenile mice as a model of pediatric TBI. Subsequently, we were interested in examining the impact of young age and sex on TBI outcome. After adapting the lateral FPI model to 21-day-old male and female mice, we characterized the molecular, histological, and functional outcomes. Whereas similar tissue injury was observed in male and female juvenile mice exposed to TBI, we observed differences in neuroinflammation and neurobehavioral function. Overall, our findings revealed less acute inflammatory cytokine expression, greater subacute microglial/macrophage accumulation, and greater neurological recovery in juvenile male mice after TBI. Given that ongoing brain development may affect progression of and recovery from TBI, juvenile models are of critical importance. The sex-dependent differences we discovered after FPI support the necessity of also including this biological variable in future TBI studies. Understanding the mechanisms underlying age- and sex-dependent differences may result in the discovery of novel therapeutic targets for TBI.

## Introduction

Traumatic brain injury (TBI) is a leading cause of death and disability that affects over 2 million people per year in the United States.^1^ Children <4 years of age have one of the highest rates of TBI and require special consideration because their nervous systems are still maturing at the time of injury.^1,2^ Clinical reports vary on how age at injury affects outcome, but several studies described that severe TBI is relatively more harmful to younger children.^3–5^ Despite the burden of TBI in young children and the unique pathophysiological features resulting from ongoing neurodevelopment, juvenile pre-clinical TBI models are underused as a means to advance treatment for this patient group.

In children, severe TBI can cause different pathoanatomical states, including focal contusion/hemorrhage and diffuse axonal injury, with most injuries being mixed. In adult rodents, an animal model of mixed injury can be induced by a lateral fluid percussion injury (FPI). Although lateral FPI has been adapted to juvenile rats, to our knowledge, it has not been used in juvenile mice, limiting the use of available transgenic and knockout lines in this mixed-TBI model.^6^

The primary aim of this study was to determine whether FPI could be used in juvenile mice to create a reproducible pediatric TBI model with characteristic tissue injury, secondary injury response, and behavioral deficits. Twenty-one-day-old mice were used to approximate the neurodevelopment of toddler-aged children. Whereas previous work was largely done in male mice, recent studies uncovered important sex differences after TBI.^7–11^ Therefore, to evaluate sex-dependent effects of juvenile TBI, we studied both males and females.

## Methods

### Animals

For our studies, 21 ± 3-day-old male and female C57BL/6J weanlings bred in-house were used. Average weight on day of craniectomy was 9.4 ± 1.5 g for males and 8.9 ± 1.3 g for females. Mice were housed in the Animal Care Facility at the University of Iowa (Iowa City, IA) under a 12-h light-dark cycle with *ad libitum* access to food and water. After craniectomy and FPI, mice remained singly caged. All procedures performed in this study were in accord with protocols approved by the Institutional Animal Care and Use Committee at the University of Iowa.

### Lateral fluid percussion injury

On the day preceding injury, mice underwent craniectomy. Mice were anesthetized with ketamine/xylazine (87 mg/kg of ketamine/12 mg/kg of xylazine) by intraperitoneal injection. The head was then mounted in a stereotactic frame, and a midline incision of the scalp was made for reflection of the skin and exposure of underlying skull. A thin (1–2 mm) disc of weed trimmer line was glued to the skull using cyanoacrylate superglue, centered between lambda and bregma sutures, and between lateral skull edge and sagittal suture. This served for stabilization of the hand-held trephine. A 3.0-mm outer diameter trephine (University of Pennsylvania Machine Shop) was used for craniectomy, but with an important modification. To prevent compression of the thin, pliable, immature skull, the handle of the trephine was removed, thereby decreasing its weight from 28 to 1.5 g. In the absence of the trephine handle, a stereotactic frame was used for stabilization of the distal end of the trephine shaft while the adherent plastic disc stabilized the proximal end, allowing for manual spinning of the trephine.

After sufficient drilling, the bone flap was gently removed by gripping the adherent plastic disc with small forceps. This creates a 3-mm craniectomy centered on the left parietal skull bone. A modified Luer-Lock hub was placed surrounding the craniectomy site and secured with cyanoacrylate glue. The hub was further secured with methyl-methacrylate dental cement (Jet Acrylic Liquid mixed with Perm Reline/Repair Resin) surrounding the bottom portion of the hub. The hub was filled with sterile saline and closed with a sterile intravenous cap to prevent exposure of the underlying dura to the environment until time of FPI. Mice recovered in a heated recovery cage until mobile. The following day, mice underwent FPI. Pendulum angle of the FPI device was adjusted before each experimental group to achieve a peak pressure between 1.1 and 1.3 atmospheres (atm) when triggered against capped intravenous tubing. For experiments in this study, the pendulum angle varied between 9.8 and 11 degrees. Mice received 3% inhaled isoflurane in an induction chamber before being transferred to a nose cone, where the intravenous cap was removed and any air bubbles in the hub were eliminated.

Once deeply anesthetized, mice were connected to the FPI device by 20-inch, 3-mm diameter intravenous tubing and placed on their right side. The pendulum was released, generating a brief fluid pulse against the exposed dura. Peak pressure of fluid pulse was measured for every FPI animal using a Tektronix digital oscilloscope (TDS460A; Tektronix, Inc., Beaverton, OR). After injury, mice were placed on their backs and their righting time was measured as an indicator of injury severity. After righting, mice were reanesthetized with isoflurane, the Luer-Lock hub was removed, and the skin incision was sutured closed. Mice receiving sham injury underwent identical treatment through connection to the FPI device. Sham mice were then disconnected without triggering the FPI device and righting reflex was measured. After skin closure, anesthesia was discontinued and animals were placed in a heated cage until recovered and ambulatory. Given that we were interested in studying moderate-to-severe TBI, FPI mice were included only if righting reflex was >5 min.^12–14^ Across all studies, the average righting time after FPI was 11.8 ± 0.5 min, which corresponded to an average peak pressure delivered of 1.29 atm.

### Real-time polymerase chain reaction

Four and 24 h after sham or FPI, juvenile mice were euthanized with isoflurane, followed by decapitation and removal of brains. Brain tissue was dissected and collected by region (left and right parietal cortex, left and right hippocampus, and brainstem) and snap-frozen on liquid nitrogen for RNA extraction; 4 h: male sham (*n* = 6), male FPI (*n* = 7), female sham (*n* = 7), and female FPI (*n* = 7); 24 h: male sham (*n* = 6), male FPI (*n* = 8), female sham (*n* = 7), and female FPI (*n* = 9–10).

Total RNA was extracted from sham or FPI brain regions using TRIzol (Invitrogen, Carlsbad, CA) as per the manufacturer's instructions. RNA yield and purity were evaluated using a NanoDrop spectrophotometer. First-strand complementary DNA (cDNA) was synthesized with SuperScript III reverse transcriptase (Invitrogen). Amplified cDNAs were diluted 1:15 in ultra-pure water and subjected to real-time polymerase chain reaction (PCR) on an Applied Biosystems Model 7900HT with TaqMan Universal PCR Mastermix (Applied Biosystems, Foster City, CA) and the following probes: interleukin (IL)-1β (Mm00434228_m1); IL-6 (Mm00446190_m1); tumor necrosis factor alpha (TNF-α; Mm00443258_m1); and glyceraldehyde 3-phosphate dehydrogenase (GAPDH; 4308313). PCR reactions were conducted as follows: 2 min at 50°C, 10 min at 95°C, followed by 40 cycles for amplification at 95°C for 15 sec and 60°C for 60 sec. Biological samples were run in duplicate or triplicate. Genes of interest were normalized to endogenous control GAPDH. Data were analyzed using the comparative cycle threshold method, and results are expressed as fold difference from sham controls.

### Immunohistochemistry

One and 21 days after sham or FPI, mice were deeply anesthetized with ketamine/xylazine and transcardially perfused with 0.9% saline, followed by 4% paraformaldehyde in 0.1 M of phosphate-buffered saline (PBS). Brains were extracted and immersed in the same fixative overnight. Brains were then paraffin-embedded, 6-μm coronal sections were cut with a rotary microtome, and sections were mounted on Plus slides. Slides were rehydrated through xylene and graded alcohols, then washed with distilled water. Antigen retrieval was performed, after which slides were cooled for 20 min at room temperature and washed twice with DAKO 1× buffer (Dako, Glostrup, Denmark). Endogenous peroxidase activity was quenched with 3% hydrogen peroxide for 8 min at room temperature, then washed twice with DAKO buffer. Blocking of non-specific antibody staining was done with avidin and biotin blocking solutions, each for 15 min.

Slides were then incubated with the following primary antibodies: 1) rabbit monoclonal anti–amyloid precursor protein (bAPP; 1:15,000, Abcam ab32136; Abcam, Cambridge, MA) for 30 min at room temperature and 2) rabbit polyclonal anti-Iba1 (ionized calcium binding adaptor molecule 1; 1:500, Wako, 019-1974; Wako Chemicals, Richmond, VA) for 60 min at room temperature. After washing, Dako Rabbit Envision was applied for 30 min, followed by Dako DAB (3,3’-diaminobenzidine) Plus for 5 min and Dako DAB enhancer for 3 min, all at room temperature. Slides were rinsed with distilled water and counterstained with hematoxylin.

### Quantification of immunohistochemistry

Automated quantification of the extent of axonal injury within regions of interest (ROIs) was performed using ImageJ software (NIH, Bethesda, MD) to calculate the proportional area with bAPP-stained axon bulbs and varicosities. An average of three sections within the lesion were analyzed for each animal, spaced 600 um apart, extending from approximately bregma −1.0 mm to −3.0 mm. The ROI analyzed was the fiber tracts running between the cortex and hippocampal formation, including the corpus callosum and external capsule. For all sections, the midline served as the medial border of the ROI. For the rostral-most section, a line drawn horizontally between the fimbria and stria terminalis and extending to the corpus callosum served as the lateral border of the ROI. For the middle section, the lateral border of the ROI was defined by a horizontal line between the hippocampal formation and the thalamus extending to the corpus callosum. Finally, for the caudal-most section, the lateral border of the ROI was defined by a horizontal line drawn from the inferior portion of the dentate gyrus to the external capsule. Images obtained with an Olympus BX-61 microscope or Olympus VS120 slide scanner (Olympus Corporation, Tokyo, Japan) were transferred to ImageJ and converted to grayscale images. Next, our ROI was outlined and manual thresholding was used to isolate the bAPP positively stained injured axons. Finally, the thresholded area within our defined ROI was measured using the analyze particles tool.

Similarly, while assessing neuroinflammation after FPI, we used automated quantification of Iba1-stained microglia/macrophages within defined ROIs. An average of three slides were used per mouse spaced 600 um apart and regions analyzed included ipsilateral cortex, axonal fiber tracts including corpus callosum and external capsule, hippocampus, and thalamus. For the cortex and corpus callosum, ROI was defined by the midline medially and extending to the same lateral borders used for corpus callosum/fiber tracts as described above. For the hippocampus and thalamus, the entire hippocampal formation and thalamus were analyzed.

### Lesion volume assessment

Sections from brains harvested at 1 and 21 days post-injury were used for analysis of lesion volume. As above, mice underwent the same process for formalin fixation and paraffin embedding. Coronal sections (6 μm) were collected every 300 μm, mounted on Plus slides, and stained with hematoxylin and eosin for further analysis. An Olympus BX-61 microscope was used to image all sections. Lesion volume was calculated using sections encompassing the lesion from its anterior to posterior border, extending from approximately bregma −1.0 mm to −3.0 mm. Using ImageJ, the area of the injured and uninjured hemispheres were measured from each serial section and used to calculate hemispheric volumes. Percent volume loss was determined by comparison of injured to uninjured hemisphere as previously reported.^15^

### Neurobehavioral testing

#### Modified Neurologic Severity Score

A composite measure of neurological function was utilized at serial time points after FPI (3, 24, and 48 h). Function was graded on a scale of 0 to 10 based on motor and sensory tests and assessment of reflexes. Points are assigned for failure to complete tasks or absent reflexes so that higher scores indicate greater deficits. Motor tests included response to tail suspension (1 point each assigned for flexion of forelimb, flexion of hindlimb, and head movement greater than 10 degrees to vertical axis) and placement on a table (1 point each assigned for inability to walk straight, circling toward paretic side, and falling to paretic side). Sensory tests included placing test (1 point assigned if no forelimb placement in response to contralateral whisker stimulation) and proprioceptive test (1 point assigned if no forelimb resistance to contralateral lateral push). Reflexes included corneal (1 point assigned if lack of eye closure in response to touch with cotton swab) and startle (1 point assigned if lack of startle with loud hand clap).^16^

#### Accelerating rotarod

Motor function was assessed using an accelerating rotarod (Columbus Instruments Rotamex-5; Columbus Instruments, Columbus, OH). The speed of the rotarod was accelerated from 4 to 40 rpm over 300 sec, with an acceleration of 1.2 rpm/10 sec. Mice underwent three trials per day with a 15-min rest interval between trials on post-injury days 1, 2, 3, and 7. Latency to fall was recorded and averaged for the three trials.

#### Barnes maze

Cognitive function was assessed using the Barnes maze. The Barnes maze consists of a gray circular table 91 cm in diameter with 20 holes, 5 cm in diameter, evenly spaced around the perimeter. The table was brightly lit and open, motivating the test subjects to learn the location of the dark escape box located under one of the 20 holes. A black curtain surrounded the Barnes maze, and four equally spaced visual cues were hung from the curtain positioned around the table. ANY-maze video tracking software (Stoelting Co., Wood Dale, IL) was used for data collection. Two weeks after sham or FPI, acquisition trials were conducted (four trials per day) for 4 days, during which time an escape box was placed under the target hole. Each trial ended when the mouse entered the target hole or after 80 sec had elapsed. Mice that did not locate the escape were guided to the target hole. All mice were allowed to remain in the escape box for 20 sec. Average latency to the escape hole was recorded for each acquisition day. On day 5 of Barnes maze testing, a probe trial was conducted to assess hippocampal-dependent spatial memory. The escape box was removed from under the target hole and mice were placed in the maze for 60 sec. Each mouse underwent one probe trial, during which the time spent in a 2-cm-diameter zone around the target hole was recorded.

### Experimental design and statistical analysis

Test animals were randomly assigned to treatment group. The tester was blinded to sex and treatment. Data are expressed as mean ± standard error of the mean (SEM). For cytokine expression, the unpaired *t*-test or Mann-Whitney U test were used depending on data distribution. For experiments requiring comparison of greater than two groups, data were analyzed by one-way analysis of variance (ANOVA) followed by Fisher's least significant difference (LSD0 or Dunn's test for multiple comparisons. For multi-day behavioral studies including Modified Neurological Severity Score (mNSS), rotarod, and training phase of the Barnes maze, analysis was by repeated-measures two-way ANOVA. Treatment group (FPI vs. sham) was the between-subjects fixed factor, time was the repeated-measure fixed factor, and subject was the random factor. The interaction of time × treatment group was analyzed as well as main effects of time and treatment. If significant effects were detected in the interaction or in the main effects, post-hoc multiple comparison testing was done using Fisher's LSD. Statistical analysis was done using GraphPad Prism software (version 7.0; GraphPad Software Inc., La Jolla, CA). A value of *p* < 0.05 was considered statistically significant.

## Results

### Experimental design

To determine whether lateral FPI could be adapted for use in juvenile mice to create a reproducible model relevant to pediatric TBI, we evaluated gene expression, neuropathology, and neurobehavioral function. Twenty-one-day-old mice were selected for use to approximate the neurodevelopment of toddler-aged children. Similar to children ages 2–3 years, the brain of a P20-21 rodent weighs approximately 90% of the adult brain and is undergoing peak synaptogenesis and myelination. Gliogenesis is also still ongoing, although beyond the peak period.^6,17^ These parallels in neurodeveloment make the P21 rodent an effective tool for the study of the unique pathophysiology of pediatric TBI and its impact on ongoing brain maturation.

**FIG. 1.**
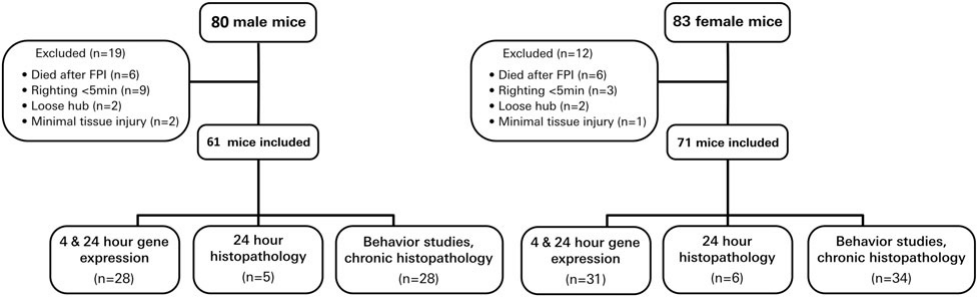
Study design. Male and female juvenile mice underwent sham or lateral FPI. Experimental endpoints included 4- and 24-h gene expression, 24-h histopathology, serial behavior studies, and 21-day histopathology. FPI, fluid percussion injury.

Our experimental design is summarized in Figure 1. Both male and female juvenile mice were studied because previous work has demonstrated sex-dependent effects after juvenile brain injury.^18,19^ Endpoints of our TBI experiments included 4- and 24-h gene expression, 24-h neuropathology, serial neurobehavioral assessment, and neuropathology at 21 days post-injury. Mortality rate was 7.5% in male mice and 7.2% in female mice after FPI. Impact seizures were common, with 85% of male mice and 80% of the female mice displaying seizure activity immediately after FPI.

### Modification of craniectomy procedure is necessary in juvenile mice

**FIG. 2.**
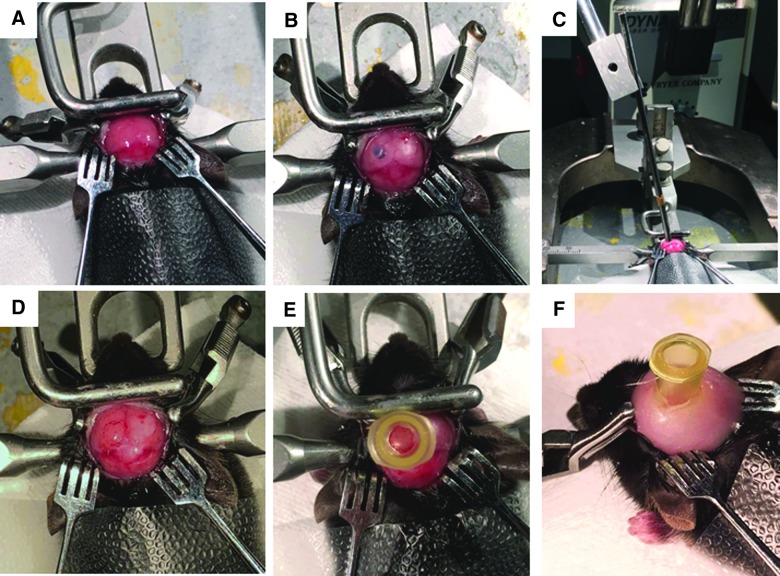
Completion of lateral craniectomy in juvenile mice using hand-held trephine. (**A**) The mouse is secured in a stereotactic frame and skin is reflected to expose the skull. (**B**) The overlying fascia is removed and a cross-section of weed trimmer line is glued centered on the left parietal skull using bregma and lambda sutures and sagittal suture and lateral skull edge. (**C**) A 3.0-mm OD trephine with handle removed is stabilized using the plastic disc and stereotactic frame while performing the craniectomy. (**D** and **E**) The bone flap is removed to expose the underlying dura, and the hub is glued surrounding the craniectomy site for attachment to the FPI device. (**F**) After the glue is dry, the hub is further secured with methyl methacrylate cement, and the injury hub is filled with saline. FPI, fluid percussion injury; OD, outer diameter.

Twenty-one ±3-day-old mice were used in our juvenile lateral FPI studies. The traditional method of craniectomy for FPI includes use of a hand-held trephine. The thin, pliable skull of juvenile mice limited the conventional use of the trephine given that its weight depresses the bone, risking injury to the underlying brain and preventing stable trephine placement. Through removal of the trephine handle, the tool was decreased in weight to 1 g and no longer caused depression of the immature mouse skull. The trephine was stabilized proximally by a plastic disc adhered to the mouse skull and distally using the arm of a stereotactic frame (Fig. 2). The operator was then able to spin the trephine manually, allowing for completion of the craniectomy. After craniectomy, the remainder of the procedure is consistent with the traditional methods used in adult FPI models and includes securing a plastic hub around the craniectomy site to be used for connection to the FPI device at time of injury.

**FIG. 3.**
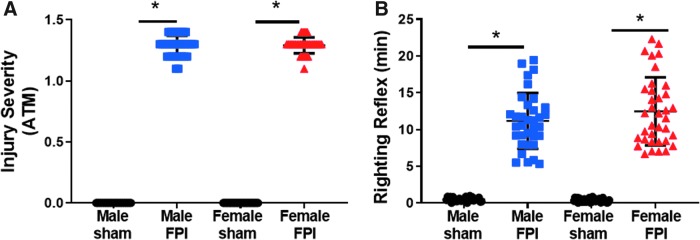
Juvenile lateral FPI results in consistent impact pressure and prolonged unconsciousness. (**A**) Peak pulse pressure delivered by lateral FPI was measured and recorded in atmospheres of pressure. (**B**) Time to right was measured for all sham and FPI subjects. Subjects across all studies included: male sham (*n* = 29), male FPI (*n* = 34), female sham (*n* = 35), and female FPI (*n* = 36). Unpaired *t*-test or Mann Whitney U test, *p < 0.05 compared to sham. ATM, atmospheres; FPI, fluid percussion injury.

As in adult FPI, a consistent impact pressure can be achieved in juvenile mice. Male and female mice were exposed to a mean pressure of 1.29 atm (Fig. 3A). This corresponded to a righting reflex of 11.17 ± 0.65 min in male mice and 12.49 ± 0.77 min in female mice (Fig. 3B).

### Juvenile lateral fluid percussion injury results in a mixed model of focal and diffuse injury

**FIG. 4.**
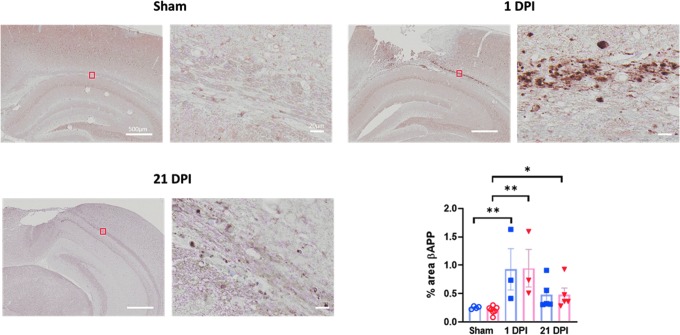
Juvenile lateral FPI results in diffuse axonal injury detectable at 1 and 21 days post-injury. bAPP immunohistochemistry was performed to assess for axonal injury at 1 and 21 days after LFPI. Images are from representative sham, 1 DPI, and 21 DPI subjects. Three to 5 mice per treatment group from at least two independent experiments were used for assessment of bAPP immunoreactivity. Data are presented as mean ± SEM. One-way ANOVA with Fisher's LSD for multiple comparisons. *p < 0.05; **p < 0.01. ANOVA, analysis of variance; bAPP, beta-amyloid precursor protein; DPI, days post-injury; FPI, fluid percussion injury; LFPI, lateral FPI; LSD, least significant difference; SEM, standard error of the mean.

Accumulating evidence indicates that pathophysiological mechanisms vary between different anatomical phenotypes of TBI. This necessitates the use of models that mirror the different TBI phenotypes for optimal translation. In children with severe TBI, combined focal and diffuse injuries are commonly observed. Lateral FPI in adult rodents has been characterized as a mixed model, generating both focal contusion and diffuse axonal injury; we would expect FPI in juvenile mice to also result in a mixed model of injury. To evaluate for diffuse axonal injury, we used immunohistochemistry staining for bAPP. bAPP accumulates in injured axons with impaired axonal transport and can be detected by immunohistochemistry. As we predicted, at 1 day post-injury, positive staining was noted in all juvenile subjects exposed to FPI, including in the corpus callosum rostrally and in the external capsule caudally. Whereas staining in the ipsilateral white matter tracts was greatest, bAPP staining of injured axons was also observed in the corpus callosum contralateral to injury in some sections. Quantification of bAPP immunostaining in the ipsilateral axonal tracts demonstrated increased staining in both male and female juvenile mice 1 day post-FPI (0.92 ± 0.36% male FPI vs. 0.25 ± 0.01% male sham, *p* = 0.01 and 0.95 ± 0.33 female FPI vs. 0.21 ± 0.03% female sham, *p* = 0.001; Fig. 4). The reported duration of bAPP accumulation after TBI varies, but with previous studies consistently demonstrating a decrease in immunostaining over time. In one previous study of controlled cortical impact, bAPP staining was no longer detectable at 28 days post-injury, whereas in a separate study of single and repetitive mild TBI (mTBI), axonal injury was still detectable at 1 year post-injury.^20,21^ On our histological analysis, bAPP staining remained detectable in most subjects at 21 days post-injury, but similarly to previous reports, with a decrease compared to at 1 day post-injury. Further, staining was predominantly perilesional by 21 days post-injury. On quantitative analysis using proportional area of staining, females showed an increase in bAPP staining at 21 days post-injury (0.48 ± 0.12% FPI vs. 0.21 ± 0.03% sham; *p* = 0.008), and males had a trend toward an increase (0.48 ± 0.26% FPI vs. 0.25 ± 0.03% sham; *p* = 0.06; Fig. 4).

Using immunohistochemistry, we also studied focal cortical tissue loss at the site of impact after FPI. Conflicting evidence exists regarding trauma induced tissue loss in juvenile models and pediatric TBI. Whereas some have reported that younger age at injury is protective from trauma-induced tissue loss, others have reported more progressive tissue loss after juvenile exposure to trauma.^22,23^

**FIG. 5.**
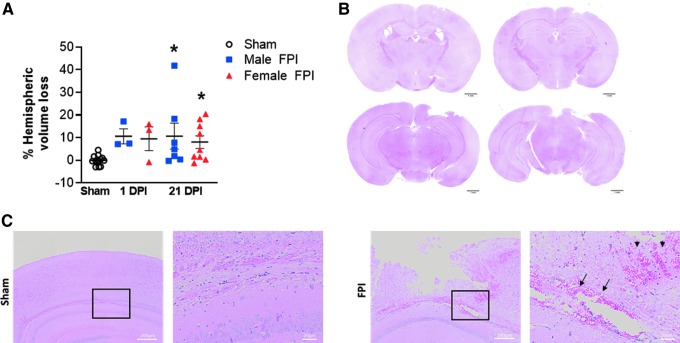
Juvenile lateral FPI results in focal, cortical tissue loss evident at 1 and 21 days post-injury. (**A**) Data are expressed as percentage hemispheric volume loss compared to contralateral, uninjured hemisphere. Data are presented as mean ± SEM. One-way ANOVA with Fisher's LSD test for multiple comparisons. *p < 0.05 compared to sham. (**B**) Representative images of H&E-stained coronal sections demonstrating tissue loss from a subject 21 days after FPI. (**C**) Representative H&E-stained sections 1 DPI demonstrating hemorrhage within the corpus callosum (arrows) and perilesional cortex (arrowheads). ANOVA, analysis of variance; DPI, days post-injury; FPI, fluid percussion injury; H&E, hematoxylin and eosin; LSD, least significant difference; SEM, standard error of the mean.

At 1 and 21 days after lateral FPI, we used hematoxylin and eosin staining to evaluate for cortical tissue loss. At 1 day post-injury, 10.6% hemispheric volume loss was observed in males and 9.5% in females (Fig. 5A). Based on the small numbers analyzed at this time point, this reached near significance (10.6 ± 3.3% male 1 day post-injury vs. −0.02 ± 0.5% sham, *p* = 0.05 and 9.5 ± 5.2% female 1 day post-injury vs. −0.0.02 ± 0.5% sham, *p* = 0.08). At 21 days post-injury, we found a similar lesion size with 12.0% volume loss in male and 10.7% volume loss in female mice exposed to FPI (12.0 ± 6.2% male FPI vs. −0.0.02 ± 0.5% sham, *p* = 0.01 and 10.68 ± 3.4% female FPI vs. −0.0.02 ± 0.5% sham, *p* = 0.03; (Fig. 5A). On histological analysis of hematoxylin and eosin–stained sections, hemorrhage was also present early after injury. At 1 day post-injury, all FPI mice were found to have blood deposition in the ipsilateral corpus callosum (Fig. 5C). This is consistent with a previous report whereby early hemorrhage in the corpus callosum distinguished juvenile mice with moderate-to-severe TBI from those exposed to mild injury.^24^

### Juvenile lateral fluid percussion injury activates inflammatory cytokine expression in focal and diffuse regions of injury

**FIG. 6.**
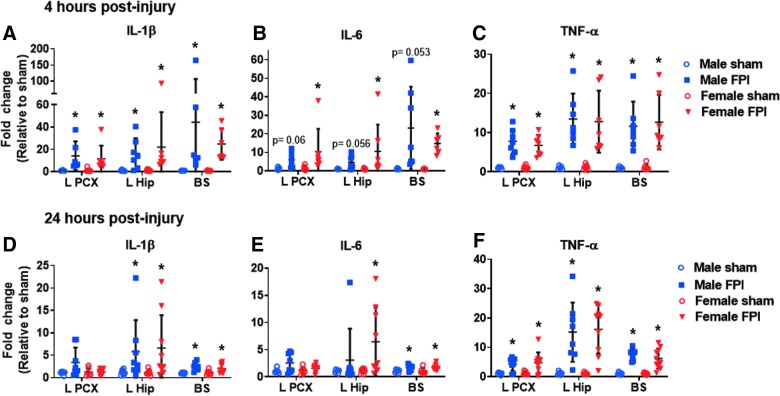
Juvenile lateral FPI elicits rapid inflammatory cytokine expression in focal and diffuse brain regions. Expression of IL-1β, IL-6, and TNF-α were evaluated by qPCR in the ipsilateral parietal cortex (L PCX), ipsilateral hippocampus (L hip), and brainstem (BS) 4 h (**A–C**) and 24 h (**D–F**) after FPI. Cytokine expression was compared in male sham (*n* = 5–6), male FPI (*n* = 6–8), female sham (*n* = 7), and female FPI (*n* = 7–10). Data are expressed as fold change in gene expression relative to sham and are presented as mean ± SEM. Unpaired *t*-test or Mann-Whitney U test depending on data distribution. *p < 0.05 compared to shams of same sex. The data are pooled from four (4 h) and three (24 h) independent experiments. FPI, fluid percussion injury; IL, interleukin; qPCR, quantitative polymerase chain reaction; SEM, standard error of the mean; TNF-α, tumor necrosis factor alpha.

Neuroinflammation is recognized as an important injury response pathway after TBI with the potential to contribute to secondary neuronal dysfunction and loss. Clinical and experimental data have indicated a robust inflammatory response to TBI in the mature brain. In our adult FPI model, we demonstrated rapid increases in cytokine expression both in areas affected by direct impact and diffuse axonal injury.^15^ In children, the potential for a dysregulated immune response after TBI to the immature brain has been reported, and as such, evaluation of the brain cytokine response after juvenile FPI was warranted.^25–27^ After lateral FPI in juvenile mice, we discovered rapid elevation in proinflammatory cytokines near the injury epicenter (cortex and underlying hippocampus) as well as in areas remote from impact (brainstem; Fig. 6). At 4 h post-injury, IL-1β and TNFα were elevated throughout the brain in both male and female juvenile mice whereas IL-6 was only significantly elevated in female mice (Fig 6A–C). By 24 h post-injury, brain cytokine expression was decreasing (Fig 6D–F).

In males and females, only TNF remained elevated in the cortex. In the hippocampus and brainstem, analyzed cytokines remained elevated in females, but at levels lower than at 4 h post-injury. In males, IL-6 was mildly elevated in the brainstem, but not in the hippocampus, and IL-1β and TNF-α remained elevated in both regions, but at levels lower than observed at 4 h.

### Rapid and persistent accumulation of microglia after juvenile lateral fluid percussion injury

**FIG. 7.**
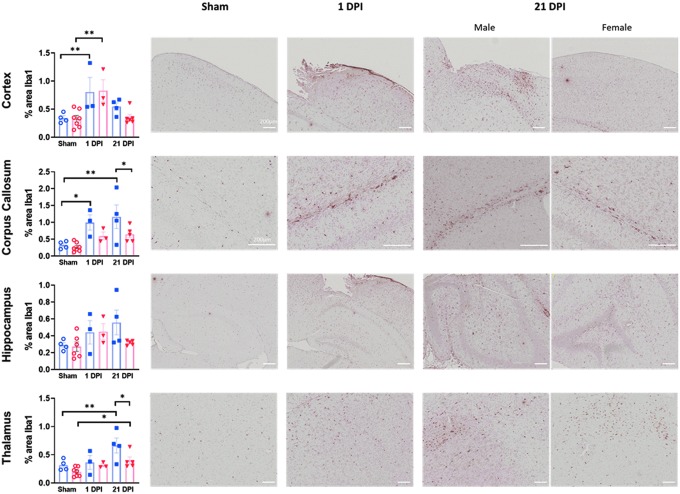
Microglia/macrophage accumulation after juvenile lateral FPI. Iba1 immunohistochemistry was performed to evaluate microglia/macrophage response to FPI. Percent area of Iba1 staining was assessed in various regions, including cortex, corpus callosum, hippocampus, and thalamus. Sex-dependent differences were present in corpus callosum and thalamus. Three to 7 mice per treatment group from at least two independent experiments for assessment of Iba1 immunoreactivity. Representative images are shown from sham, 1 DPI male, and 21 DPI male and female subjects. Data are presented as mean ± SEM. One-way ANOVA with Fisher's LSD for multiple comparisons. *p < 0.05; **p < 0.01. ANOVA, analysis of variance; DPI, days post-injury; FPI, fluid percussion injury; Iba1, ionized calcium binding adaptor molecule 1; LSD, least significant difference; SEM, standard error of the mean.

In addition to analyzing the tissue expression of cytokine levels, we sought to better characterize the cellular immune response to juvenile FPI. Microglia are the primary resident immune cells within the brain and, after a neurological insult, rapidly accumulate and activate in injured tissue. Whereas microglia play a key role in the clearance of debris and other reparative processes after injury, there is also evidence that activated microglia may cause further damage. Using immunohistochemistry to detect Iba1-positive microglia and macrophages, we investigated the response of microglia/macrophages to lateral FPI in male and female juvenile mice. We quantified the area of Iba1 staining in several injured brain regions at two time points post-injury. In the cortex, the tissue directly impacted by FPI, we found that the Iba1-stained area was increased in both males and females at 1 day post-injury, but had resolved by 21 days post-injury (Fig. 7).

In the corpus callosum, a region affected predominantly by axonal injury, sex-based differences in the microglial/macrophage response were noted. In males, a rapid accumulation of microglia/macrophages was again noted with increased Iba1 staining at 1 day post-injury (1.00 ± 0.23% FPI vs. 0.33 ± 0.06% sham; *p* = 0.02). Further, injury-induced microglia/macrophage accumulation continued at 21 days post-injury in males (1.17 ± 0.35% vs. 0.33 ± 0.06% sham; *p* = 0.002) and was significantly higher than the Iba1 staining observed in FPI females at this time point (1.17 ± 0.35% male FPI vs. 0.64 ± 0.10% female FPI; *p* = 0.03). In females, there was no increase beyond sham levels at either time point assessed, although there was a trend toward an increase at 21 days post-injury (0.64 ± 0.10% 21 days post-injury FPI vs. 0.26 ± 0.05% sham; *p* = 0.06).

In the hippocampus, whereas activated microglia were apparent on immunohistochemistry in FPI-injured mice, the area of Iba1 staining was not significantly different in any of the FPI and sham groups. Finally, similar to the corpus callosum, in the thalamus, sex-dependent differences in Iba1 staining were again noted. Whereas both males and females showed greater thalamic Iba1 staining at 21 days post-injury compared to shams (0.66 ± 0.13% male FPI vs. 0.32 ± 0.05% male sham, *p* = 0.004 and 0.40 ± 0.06% female FPI vs. 0.20 ± 0.03% female sham, *p* = 0.03), FPI males had greater Iba1 staining compared to FPI females at this time point (0.66 ± 0.13% male FPI vs. 0.40 ± 0.06% female FPI; *p* = 0.02).

### Juvenile lateral fluid percussion injury results in neurobehavioral dysfunction

**FIG. 8.**
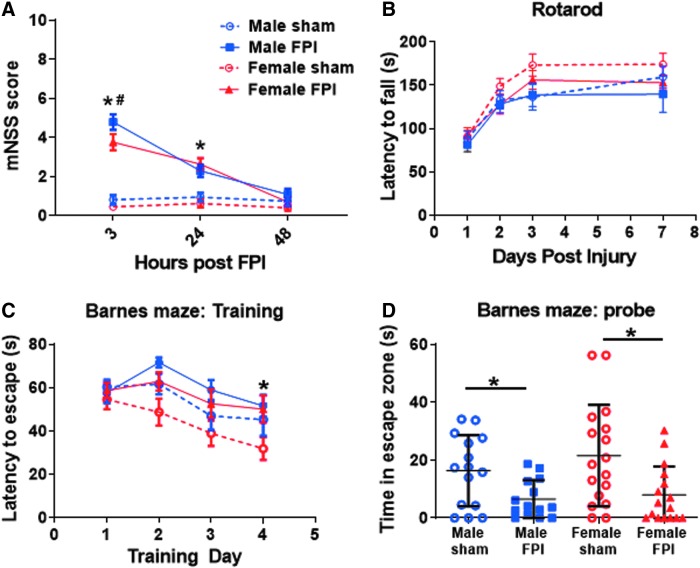
Neurobehavioral dysfunction after juvenile lateral FPI. (**A**) mNSS testing of neurological dysfunction at serial time points after FPI in male and female juvenile mice. Two-way RM ANOVA showed a significant interaction between time and treatment group (*F*_(6,118)_ = 18.68; *p* < 0.0001) and post-hoc analysis revealed worse performance by both sexes of FPI subjects compared to their respective sham groups at 3 and 24 h post-injury (*p* < 0.0001, at 3 h for both male and female FPI vs. respective same-sex sham groups; *p* = 0.0008, male FPI vs. male sham at 24 h; and *p* < 0.0001, female FPI vs. female sham at 24 h post-FPI). Post-hoc analysis also revealed worse performance in male FPI subjects compared to female FPI subjects 3 h post-injury (*p* = 0.008). Two-way RM ANOVA, post-hoc analysis by Fisher's LSD (*p < 0.05, FPI vs. sham group of same sex; ^#^*p* < 0.05, male vs. female FPI). (**B**) Accelerating rotarod showing latency to fall as a measure of sensorimotor dysfunction. The main effect of time was significant (*F*_(3,189)_ = 46.24; *p* < 0.0001), but there was no interaction of time and treatment and no significant difference between injured and sham groups. Two-way RM ANOVA, post-hoc analysis by Fisher's LSD. (**C**) Barnes maze training showing latency to identify escape hole as an indication of learning ability. There was no significant interaction between time and treatment, but main effects of time (*F*_(3,232)_ = 8.069; *p* < 0.0001) and treatment (*F*_(3,232)_ = 7.104; *p* = 0.0001) were significant. Post-hoc analysis revealed impaired learning in female FPI versus female sham subjects on day 4 of training (*p* = 0.01). Two-way RM ANOVA, post-hoc analysis by Fisher's LSD (*p < 0.05, female sham vs. female FPI). (**D**) Barnes maze probe trial showing time spent in the escape zone as a measure of hippocampal-dependent memory. One-way ANOVA with Fisher's LSD for multiple comparisons. Impaired memory in male FPI versus male sham (*p* = 0.04) and female FPI versus female sham (*p* = 0.003). *n* = 14–17 mice per group for all of the above behavior experiments. The data are pooled from eight independent experiments. FPI, fluid percussion injury; LSD, least significant difference; mNSS, Modified Neurological Severity Score; RM ANOVA, repeated-measure analysis of variance.

TBI is a leading cause of neurological dysfunction in children. After severe TBI, many children suffer persistent cognitive, emotional, and social impairments resulting in dramatic effects on quality of life. As such, the ability to detect neurobehavioral dysfunction in experimental models of juvenile TBI is imperative. After exposure of male and female juvenile mice to sham or FPI procedures, cohorts of mice underwent serial functional testing (Fig. 8). This included a composite assessment of neurological function early after TBI using a modified neurologic severity score. Serial mNSS testing at 3, 24, and 48 h after FPI revealed the greatest neurological dysfunction acutely after injury, with resolution by 48 h. At 3 h post-FPI, both male and female FPI subjects performed significantly worse than shams of the same sex (mean mNSS male FPI = 4.79 vs. male sham = 0.8, *p* < 0.0001; female FPI = 3.75 vs. female sham = 0.44, *p* < 0.0001).

Of note, male FPI mice showed greater dysfunction than female FPI mice at this time point (mNSS male FPI = 4.79 vs. female FPI = 3.75; *p* = 0.008). At 24 h, milder neurological dysfunction continued in FPI subjects and there was no longer a sex difference (mean mNSS male FPI = 2.29 vs. male sham = 0.93, *p* = 0.0008; female FPI = 2.63 vs. female sham = 0.61, *p* < 0.0001; male FPI = 2.29 vs. female FPI = 2.63, *p* = 0.38). By 48 h post-injury, there was no difference between sham and FPI subjects in mNSS (Fig. 8A).

Mice also underwent sensorimotor assessment using the accelerating rotarod on days 1 through 3 and 7 post-injury. Despite the mild motor deficits noted on day 1 in the motor component of the mNSS, juvenile mice did not show any impairment on the accelerating rotarod (Fig. 8B).

In order to assess for TBI-induced cognitive dysfunction, Barnes maze testing was conducted in juvenile FPI and sham-injured mice. Testing was initiated on day 13 post-injury and extended through day 18. During the first 4 days of Barnes maze training, learning was assessed. On the fifth day of testing, hippocampal-dependent memory was evaluated through use of a probe trial. For both sexes, there was no interaction between time and treatment during the training phase (*F*_(9,232)_ = 0.5882; *p* = 0.81). However, the main effects of time and treatment were significant (*F*_(3,232)_ = 8.069; *p* < 0.0001 and *F*_(3,232)_ = 7.104; *p* = 0.0001). Post-hoc multiple comparisons revealed a sex-dependent effect of TBI on learning given that only female FPI mice performed worse than sham mice on day 4 of training (latency to escape female FPI = 31.81 sec vs. female sham = 18.23 sec; p = 0.01; Fig. 8C). In the probe trial, both male and female juvenile mice showed TBI-induced hippocampal dependent memory impairment (time in escape zone male FPI = 6.5 sec vs. male sham = 16.35 sec, *p* = 0.04; female FPI = 7.97 sec vs. female sham = 21.54 sec; *p* = 0.003; Fig 8D).

## Discussion

Mounting evidence indicates age-dependent differences in the pathophysiology of neurological diseases necessitating increased use of immature animals when modeling pediatric TBI. While several models of TBI have been developed in juvenile rats, to date, only the controlled cortical impact model has been reported in juvenile mice. The further development of murine juvenile TBI models is of great importance given the role for genomic manipulation in dissecting TBI pathophysiology. Further, given that lateral FPI is a mixed focal and diffuse injury model, it may have greater translatability for pediatric TBI, warranting its development in juvenile mice. The technical difficulty of performing craniectomy on immature mice has likely been a major deterrent to the development of the murine juvenile lateral FPI model. With the simple innovation of removing the handle from the trephine to decrease its weight followed by stabilization of the trephine with a stereotactic frame, craniectomy of the soft, immature mouse skull became easily achievable.

Following this modification to the standard craniectomy procedure, we successfully adapted lateral FPI to 21-day-old juvenile mice. We demonstrated that murine juvenile lateral FPI results in diffuse axonal injury, focal tissue injury, neuroinflammation, and neurobehavioral dysfunction. Combining this mixed injury model with transgenic and knockout mice will further our ability to determine the biological basis of age-dependent differences in TBI outcome and dissect the pathophysiology of pediatric TBI.

In addition to developing and characterizing a new model of pediatric TBI, a further aim of our study was to assess the impact of sex on juvenile FPI. Whereas sex-dependent differences in the pathophysiology of TBI are recognized, this has received greater study in adults because of an emphasis on the impact of post-pubertal sex steroids on TBI pathogenesis. However, it is well recognized that early sex-based differences in the brain exist and may be attributable to the gonadal steroidogenesis that occurs in the late fetal period as well as differences in genes expressed by sex chromosomes. Increasingly, a number of neurodevelopmental processes have been found to have sex-based differences, yet the impact of these differences on secondary injury after TBI remains understudied.^18,28^

In our study, we investigated two different aspects of the neuroinflammatory response to juvenile FPI and found sex-based differences in both. The neuroinflammatory environment after pediatric TBI is of particular interest, given that during development, microglia exist in a more activated stated attributable to their role in synaptic pruning and phagocytosis. Further, sex-based differences in gliogenesis have been demonstrated, including a previous study showing greater numbers of activated microglia in male mice at a very young age (P4) with a switch to greater microglial numbers in female mice by P30.^29^ In our study, we found that male mice showed greater accumulation of microglia/macrophages after juvenile FPI, particularly in areas affected by axonal injury and at delayed time points post-injury.

Though limited study has been done on the impact of sex on neuroinflammation after pediatric TBI, a previous study of repetitive mTBI in young rats also found greater Iba1 immunoreactivity in males.^30^ Conversely, we found that female mice had greater elevations in IL-6 expression at both 4 and 24 h after injury. Given that this was tissue-based cytokine expression, the cell type responsible for this difference is unknown. Further evaluation of the impact of sex on brain cytokine levels after pediatric TBI will be required. Additionally, because astrocytes, endothelial cells, and infiltrating peripheral immune cells also contribute to neuroinflammation, the impact of sex on these cell types after pediatric TBI will be required in future studies.

Besides impacts on neuroinflammation, we also found sex differences in functional outcomes. Very early after FPI, male mice had higher neurological severity scores whereas 2 weeks after injury, only female mice displayed impaired learning in the Barnes maze. It is unclear whether differences in neuroinflammation are related to differences in functional recovery after FPI, and this will be further investigated in future studies. These findings, however, are consistent with the growing work demonstrating sex differences in response to pediatric TBI. In a large, retrospective, clinical trial of pediatric TBI, girls had a longer intensive care unit length of stay and a trend toward worse functional outcome, particularly in older children (ages13–19). In studies of mTBI/concussion, girls were also shown to have longer duration of symptoms.^4,31^ After moderate-severe experimental TBI, a recent study demonstrated sex differences in adult social dysfunction after juvenile TBI.^19^ Additionally, in several models of mild juvenile TBI, sex-dependent differences were observed in social, cognitive, and electrophysiological functions.^32–34^ Our results, along with these studies, highlight the need for inclusion of sex as a biological variable in ongoing pediatric TBI studies.

Whether the developing brain is more resilient or susceptible to traumatic injury is an area of uncertainty. Although several studies have reported the highest mortality in young children (<2–7 years), a recent prospective trial of pediatric severe TBI demonstrated no difference in mortality across three different age strata.^5,35–37^ In pre-clinical TBI studies, there has also been variability in the reported outcomes. Following models of moderate-to-severe TBI, separate studies have reported greater, less, and equivalent tissue loss in juvenile compared to adult mice.^22,23,38^ A recent study comparing functional outcomes after controlled cortical impact in juvenile and adult mice reported less motor and cognitive dysfunction in juvenile TBI mice.^22^ This lack of consensus highlights the urgent need for the development of additional models of pediatric TBI and further research studying the impact of age on TBI outcome. Given that the primary aim of this study was to characterize a new model of juvenile FPI in mice, concurrent experiments in juvenile and adult mice were not done. However, through the comparison of our results in these juvenile FPI studies to our previous studies in adult FPI, several differences are worth noting.

In the current study of lateral FPI, we found that juvenile mice had similar neuropathology to that observed in our earlier studies of adult mice, including a comparable degree of cortical tissue loss and the presence of axonal injury after TBI. However, compared to our previous studies of adult FPI, cytokine expression in juveniles was lower at 24 h post-injury, consistent with more rapid resolution of inflammatory cytokines.^15^ Additionally, juvenile male mice also had less neurological dysfunction in the subacute phase compared to adult male mice after lateral FPI. This included absence of sensorimotor impairment on rotarod testing and absent learning impairment in Barnes maze training. Because these differences are based on comparisons to past studies, we are unable to control for other experimental factors that may have unintentionally changed over time. We therefore cannot definitively determine whether outcome differences are attributable to age at injury. These outcome differences are of great interest, however, and will be investigated in future studies designed specifically to evaluate the impact of age on TBI.

In recent years, public awareness of the scope of TBI has improved and there has been a dramatic increase in TBI research and funding, leading some to call the current era the golden age of TBI research.^39^ Despite this, there has still been a failure to translate novel, effective treatments. One explanation is inadequate pre-clinical modeling, including insufficient attention to relevant biological factors such as age and sex. In this study, we have confirmed age- and sex-dependent differences after murine TBI. Ongoing work will dissect the mechanisms by which these differences in secondary inflammation and neurological dysfunction occur. This may ultimately assist with the identification of effective therapies in targeted populations after TBI.
